# Is soleus intrinsic motor neuron excitability contributing to motor deficits in runners with Achilles tendinopathy?

**DOI:** 10.1007/s00421-025-05824-z

**Published:** 2025-06-01

**Authors:** Gabriel L. Fernandes, Lucas B. R. Orssatto, Gabriel S. Trajano

**Affiliations:** 1 Physical Activity, Sport and Exercise Research (PASER) theme, Faculty of Health, Southern Cross University, Gold Coast, Australia; 2 School of Exercise and Nutrition Sciences, Faculty of Health, Queensland University of Technology (QUT), Brisbane, Australia; 3 Centre for Sensorimotor Performance, School of Human Movement and Nutrition Sciences, The University of Queensland, Brisbane, QLD Australia

**Keywords:** Achilles tendon, Running, Motor unit, Persistent inward current, Triceps surae, Neuromodulation

## Abstract

**Objective:**

Soleus weakness is suggested to contribute to Achilles tendinopathy (AT) in runners. Since muscle force relies on the ability of motor units firing at high frequencies, and intrinsic motor neuron excitability contributes to firing rate modulation, soleus inhibition and hypoexcitability may contribute to AT soleus weakness. This study investigated soleus motor neuron excitability by comparing: (i) estimates of persistent inward currents (Δ*f* and Δ*f*/*k*), which is known to modulate excitability; (ii) brace height, which is related to neuromodulatory input onto motor neurons; (iii) attenuation slopes, which estimate the influence of inhibitory input onto the motor units; and (iv) and motor unit firing rates between runners with and without mid-portion AT.

**Methods:**

Delta frequency absolute and normalized (Δ*F* and Δ*F*/*k*), brace height, attenuation slope, and peak firing rates were compared between runners with AT (*n* = 11) and without AT (*n* = 12). These variables were calculated from ramp triangular-shaped isometric plantar flexor contractions at 20% maximal torque. Soleus motor unit firing rates were assessed using high-density surface electromyography.

**Results:**

No significant differences were found between groups in soleus Δ*F* (estimated mean difference: − 0.1 pps; 95% CI: − 1.3 to 1.0; *p* = 0.79), Δ*F*/*k* (0.2 pps; − 0.03 to 0.3; *p* = 0.09), brace height (− 3.9% rTri; − 8.3 to 0.4; *p* = 0.07), attenuation (0.04 pps/% torque; − 0.04 to 0.1; *p* = 0.27), or peak firing rates (− 0.4 pps; − 1.6 to 0.9; *p* = 0.55).

**Conclusion:**

Soleus motor neuron excitability or firing rates may not contribute to plantar flexor weakness in AT. Future studies should investigate other neurophysiological mechanisms and gastrocnemius contributions to AT-related weakness.

## Introduction

Achilles tendinopathy (AT) is a painful condition affecting the Achilles tendon, often becoming chronic, and significantly affecting muscle–tendon function (Rio et al. 2014). As an overloading injury, the etiology of AT is multifactorial; however, deficits in muscle performance are considered key contributing factors (O’Neill et al. 2019; Mahieu et al. 2006), which may persist long after symptomatic recovery (Silbernagel et al. 2007). Soleus dysfunction has been appointed as the primary factor influencing the plantarflexor strength and endurance deficits observed in runners with AT (O’Neill et al. 2019). Soleus selective weakness (Hug and Tucker 2017) may lead to uneven loading to the Achilles tendon, potentially contributing to tendon pain, pathology, and progression of chronic AT (Cook and Purdam 2009). Thus, understanding the neuromuscular factors influencing muscle deficits in in AT may assist with developing effective rehabilitation strategies.

From a neurophysiological perspective, deficits in soleus force in AT could be a result of a reduced ability to recruit motor units and/or an inability to modulate motor neuron firing (Enoka and Duchateau 2017). Motor neurons play a crucial role in integrating excitatory and inhibitory inputs, and accelerating, amplifying and prolonging its firing output to generate appropriate motor output (Orssatto et al. 2021). This input–output gain system is facilitated by the generation of persistent inward currents (PICs) largely within the motor neuron dendrites (Binder et al. 2020). This amplification system is essential for normal muscle function, and an inability to produce PICs can significantly reduce a muscle’s capacity to generate force (Trajano et al. 2020; Heckman et al. 2005). PICs are highly sensitive to localized inhibitory inputs, including reciprocal (Orssatto et al. 2021; Hyngstrom et al. 2008) and recurrent inhibition (Vandenberk and Kalmar 2014) which reduce motor neuron excitability and have great impact in the discharge rates of low-threshold motor unit (Hug et al. 2024). Pain associated with tendinopathies (Cook et al. 2016) activates spinal inhibitory circuits via descending pathways, which may suppress PICs (Sohn et al. 2000; Farina et al. 2004). Given the importance of PICs for normal muscle function, this increased inhibition observed in AT could at least partially explain their impaired muscle function. Such impairment may affect strain distribution through the Achilles tendon, contributing to AT etiology of tendinopathy (Trajano et al. 2020; Heckman et al. 2005; Cook et al. 2016; Millar et al. 2021).

PIC’s contribution to motor neuron firing is estimated in humans by calculating the recruitment–derecruitment hysteresis (delta frequency—Δ*F*) calculated through the paired-motor unit technique (Trajano et al. 2020; Gorassini et al. 2002). Δ*F* is the difference in the firing rate of a lower-threshold control unit at the time of recruitment and de-recruitment of a higher-threshold test unit (Trajano et al. 2020; Gorassini et al. 2002). Δ*F* provides an estimate of the contribution of PICs to the self-sustained firing of motor neurons (Gorassini et al. 2002) and is known to be influenced either by changes in neuromodulatory and inhibitory inputs to the motor neuron. Recently, two different metrics have been developed with the intention of discriminating neuromodulatory and inhibitory effects on PICs and its influence on motor neuron discharge behavior: (i) the brace height, which quantifies the deviation from linearity of the ascending portion of motor unit firing rates, serves as an indicator of neuromodulatory influence of PICs on motor unit firing patterns; and (ii) the attenuation slope has been shown to be associated with inhibitory input onto the motor neurons. Given previous suggestions of selective soleus weakness in Achilles tendinopathy (O’Neill et al. 2019), this study aimed to investigate the contribution of PICs as a potential mechanism affecting soleus motor unit firing rates in this population. To do this, we compared soleus Δ*F*, brace height, and attenuation slopes in runners with and without mid-portion AT. We hypothesized that runners with AT would exhibit lower Δ*F* amplitude and brace height, higher attenuation slopes values, as well as lower motor unit mean firing rates compared to runners without AT.

## Methods

Twenty-three endurance runners with (*n* = 11, 6 males, 45.5 ± 11.7 years old, 172.8 ± 9.5 cm, 77.2 ± 15.0 kg, and without AT (*n* = 12, 7 males, 33.0 ± 6.4 years old, 170.9 ± 8.7 cm, 65.6 ± 11.9 kg) were recruited from running clubs in Southeast Queensland, Australia. All participants had been running more than twice weekly for over 4 months. The AT group averaged 39.4 ± 16.6 km/week, while the control group, 29.1 ± 13.3 km/week. These runners also participated in a previous study from our laboratory (Fernandes et al. 2021; Fernandes et al. November 2022) where strength and endurance values were reported.

Diagnosis of mid-portion AT was confirmed by an experienced physiotherapist (GLF) during examination, based on the following criteria: (a) localized mid-portion Achilles tendon pain lasting more than 3 months, (b) pain provoked by physical activities that load the Achilles tendon in a dose dependent manner, and (c) tenderness upon palpation at the mid-portion of tendon. Volunteers were excluded if they had insertional AT; a history of Achilles tendon rupture or surgery; clinical findings indicating differential diagnosis for the Achilles tendon pain (such as tendon tear); regular participation in high-speed running sports (e.g., football, rugby, AFL), VISA-A score > 90 points for the AT group, or < 100 for the control group. Additionally, participants were excluded for any current musculoskeletal injuries of the lower limb, neurological disorder, or mental health issues affecting consent. All participants reported no comorbidities such as cardiac, pulmonary, renal, endocrine or gastrointestinal disease and were not taking any medication that could affect tendon structure (Knobloch et al. 2016). Prior to testing, all participants read and signed a detailed informed consent document and completed the VISA-A questionnaire (Martin et al. 2018). The average VISA-A score for the AT group was 69.2 ± 9.7 and 100 for the control group. This study was approved by the Queensland University of Technology Human Research and Ethics Committee in accordance with the Declaration of Helsinki.

### Torque acquisition

Plantar flexor isometric peak torque was assessed using an isokinetic dynamometer (Biodex Medical Systems, Shirley, New York). For the bilateral AT presentations (*n* = 3), the most symptomatic leg was tested, while the dominant leg was used for the control group. Leg dominance was determined by asking the participants which leg they would use to kick a ball. Participants were positioned seated at 75° of hip flexion, with their knee straight and ankle at 90°. The warm-up consisted of 2 × 4 s isometric contractions at perceived intensities of 20%, 40%, 60% and 80% of their maximal voluntary isometric contraction. After warming up, participants performed at least three maximal voluntary isometric contractions, with the highest values recorded once less than 5% variation was observed between contractions. Subsequently, participants were familiarized with triangular-shaped contractions at 20% of their peak torque, a method extensively utilized for calculating ΔF using the paired motor unit technique (Trajano et al. 2020; Hassan et al. 2020). Each participants had four attempts to practice the task, with approximately 30-s rest between contractions before recordings commenced. The rate of torque rise, and decline was standardized at 2% peak torque/s (10-s up and 10-s down), followed by a 1-min rest between contractions. Participants received real-time visual feedback of the triangular pathway, displayed on a monitor. Trials that did not closely follow the torque trajectory were excluded and repeated, resulting in two successful contractions recorded for each participant.

### Surface electromyography recordings

During the plantar flexor triangular-shaped contractions, torque and high-density surface electromyography (HD-EMG) signals were recorded from the soleus using OT Biolab+ software (version 1.3.0., OTBioelettronica, Torino, Italy). Electrodes were positioned according to the estimated muscle fiber orientation. A bi-adhesive layer with a conductive paste was used to ensure optimal skin–electrode contact and conductivity. Two 32-channels electrode arrays (ELSCH032NM6, OTBioelettronica, Torino, Italy) were placed on soleus—one laterally and one medially to the Achilles tendon. The use of two electrode arrays aimed to increase the number of identified motor units. Data from both electrodes were combined into a single file to increase motor unit yield prior to analyzing the firing characteristics of the soleus motor unit. The ground strap electrode (WS2, OTBioelettronica, Torino, Italy) was dampened and secured at the level of the malleoli of the tested leg. The EMG signals were recorded in monopolar mode, amplified (256 ×), band-passed filtered (10–500 Hz), and converted to a digital signal at a sampling rate of 2048 Hz using a 16-bit wireless amplifier (Sessantaquattro, OTBioelettronica, Torino, Italy) before being stored for offline analysis.

### Motor unit identification

HD-EMG signals from the three contractions performed by each participant were analyzed offline. These signals were decomposed into motor unit spike trains and converted into instantaneous firing rates with a specialized software using blind source separation decomposition technique, DEMUSE tool (v.4.1; The University of Maribor, Slovenia) (Vecchio et al. 2020). Motor units were across the two best contractions and all motor units underwent visual inspection by GLF. Erroneous firing times were excluded while missed firings were manually included (Vecchio et al. 2020). Manual editing and visual inspection are essential for reducing automatic decomposition errors and improve data reliability (Martinez-Valdes et al. 2016). Only motor units with a pulse-to-noise ratio > 30 dB were considered for data analysis (Vecchio et al. 2019).

### Δ*F* and peak firing rates

**Fig. 1:**
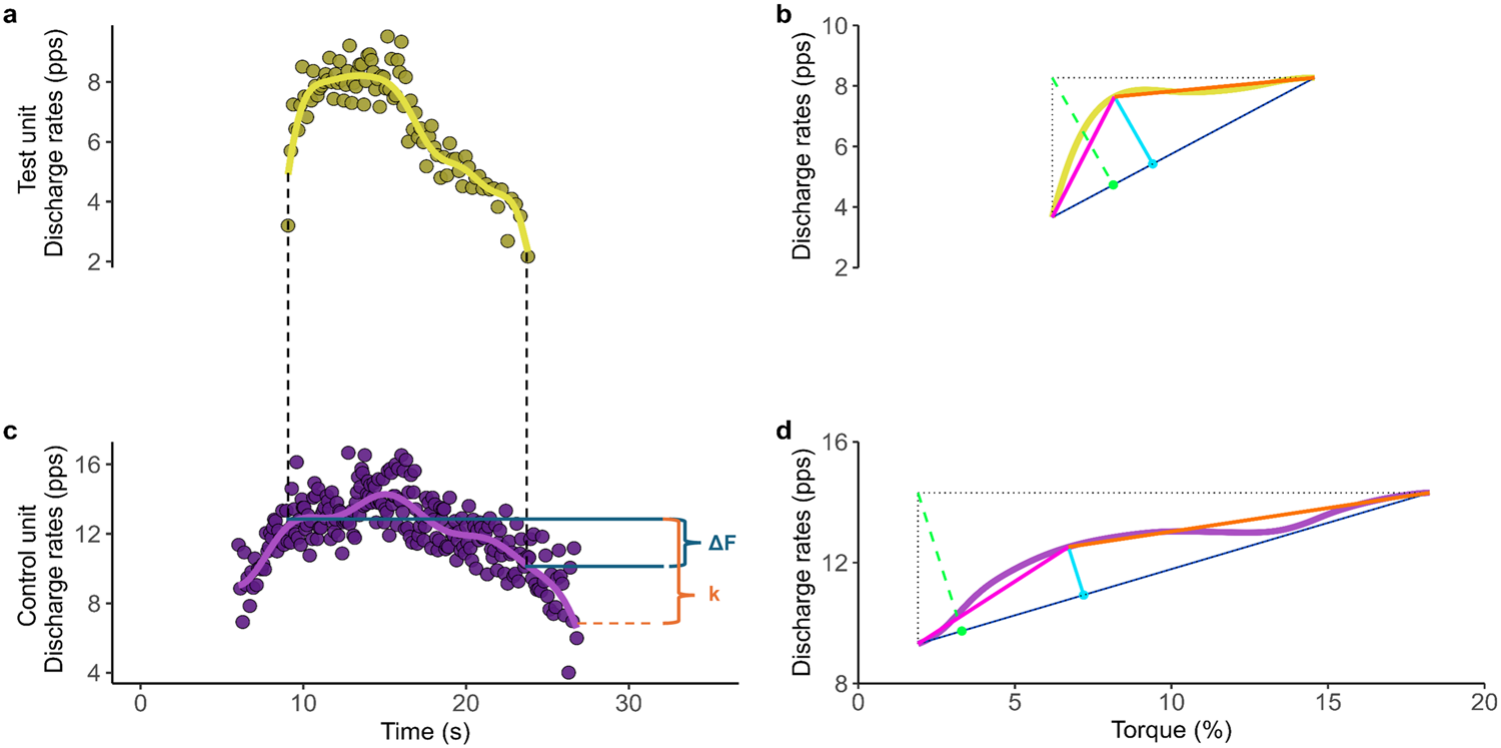
Data demonstrating the paired motor unit method for measuring delta frequency (Δ*F*) from a single participant during a triangular-shaped contraction reaching 20% of peak isometric torque. Panels **a**, **c** motor unit firing rate for a test unit (**a**) and a control unit (**b**). The yellow (**a**, **b**) and light purple (**c**, **d**) continuous line, generated using support vector regression (SVR), represent estimates of motor unit firing frequencies fitting to the instantaneous firing rate of the respective motor unit. The area in between the blue solid lines indicates the estimated amplitude of PICs (ΔF) and the area between the orange dotted lines represent *k* (**c**). Panels (**b**, **d**) brace height calculation are shown as a light blue solid line; with brace height normalized by the right triangle (%rTri) demonstrated as light green dotted line. The pink line illustrates the acceleration slope while the orange line represents the attenuation slope. *pps* pulses per second

Firing events for each motor unit were converted into instantaneous firing rates, which were then smoothed using support vector regression (SVR) to provide a continuous estimate of firing rates for each motor unit (Beauchamp et al. 2022). The maximum value obtained from the SVR curve was considered the peak firing rate. The recruitment threshold was considered as the relative torque (%) produced at the time each motor unit was recruited and was used to characterize the populations of motor units identified by the decomposition algorithm for each group. PIC amplitude was estimated using the paired motor unit analysis (Gorassini et al. 2002) (Fig. 1a, b). In this method, motor units with a lower recruitment threshold (control units) were paired with those exhibiting higher recruitment threshold (test units). The change in firing rates of the control motor unit from the moment of recruitment to the de-recruitment of the test unit was calculated as Δ*F* (Heckman et al. 2005; Gorassini et al. 2002). Pairs of motor units were generated using the following criteria: (1) rate-to-rate correlation between the test and the control units with an *r* value of ≥ 0.7; (2) test units was recruited 1.0 s after the control units; and (3) the control unit showed no firing rate saturation following the recruitment of the test unit (i.e., the firing rate of the control unit at the time of test unit recruitment, minus the peak firing rate at the control unit > 0.5 pps) (Vandenberk and Kalmar 2014; Gorassini et al. 2002). The Δ*F*s obtained for each control unit were averaged to obtain a single Δ*F* for each test motor unit. Additionally, Δ*F* was normalized by a factor *k* (Škarabot et al. 2023), defined as the theoretical maximal firing rate hysteresis of the control unit. Specifically, (*k*) was computed as the difference between the smoothed discharge rate (via SVR) of the control unit at the recruitment time of the test unit and the smoothed discharge rate of the control unit at derecruitment. This value was used to normalize to normalize Δ*F* within each motor unit pair. Thus, the Δ*F*/*k* ratio quantifies the degree to which the test unit relies on persistent inward currents (PICs) to sustain firing after synaptic input has decreased. Motor unit firing frequencies were determined based on the first two discharge times at both recruitment and derecruitment. Additionally, the time from recruitment-to-peak firing rate (ascending time), the time from peak firing rate to derecruitment (descending time), and the ratio of ascending to descending firing time were calculated and compared between groups.

### Brace height and attenuation slope

Brace height is a geometric measure used to investigate the neuromodulatory effect of PICs on motor neuron firing patterns. It quantifies the largest deviation in firing rate (in this case the SVR curve plotted as a function of torque) from a hypothetical linear progression, extending from motor unit recruitment-to-peak firing rate. A linear reference is drawn between the initial recruitment and peak firing rates. Brace height is the maximum orthogonal distance from this reference line and the smoothed firing trace. Brace height is then normalized as a percentage of the height of a theoretical right triangle (rTri%), where the hypotenuse represents highest orthogonal distance possible from linear progression from recruitment-to-peak firing. In addition to brace height, the attenuation slope was calculated as an estimation of the inhibitory effect on PICs (Beauchamp et al. 2023). A slope was calculated between the point from which the brace height was calculated and the maximum value of the SVR curve (Fig. 1b, d).

### Statistical analysis

Separate linear mixed-effect models were used to compare soleus Δ*F*, Δ*F*/*k*, brace height, attenuation slope, peak firing frequency, firing frequencies at recruitment and at de-recruitment, time from recruitment-to-peak firing rate (ascending time), time from peak firing rate to de-recruitment (descending time) and ascending/descending firing time ratio between groups. Since age is a factor that can influence the estimates of PICs (Orssatto et al. 2021; Orssatto and Blazevich 2023), and the AT group was significantly older than the control group, a random intercept for each participant (accounting for age) was considered in the ΔF model to control for the influence of age and the correlation between repeated observations (clusters of motor units) within each participant (Boccia et al. 2019). The recruitment-to-peak firing rate modulation was included as random effect in the brace height and attenuation slope models. The final models were selected from a series of candidate models, based on the smallest Bayesian Information Criterion (BIC) and Akaike Information Criterion (AIC) values. When a significant effect was observed, pairwise comparisons were used. Statistical significance was accepted at *p* ≤ 0.05 for all tests. Data are presented as mean ± SD or mean (95% confidence interval lower to upper limits) as reported. Models were fitted using the *lme4* and *lmerTest* package (Bates et al. 2015). Estimated marginal mean difference and 95% confidence intervals (CI) for Δ*F*, peak firing rate, and recruitment threshold between groups were determined using the *emmeans* package (Lenth 2016). Hedges’ g effect sizes are reported for all variables, with values < 0.2 small, 0.5–0.7 medium, and > 0.8 large (Lakens et al. 2013). All statistical analyses were undertaken using R studio (version 1.3.1093).

## Results

A total of 235 spike trains were recorded for the AT group, with a median of 18 spike trains per participant (25th percentile: 10, 75th percentile: 33). For the control group, 207 spike trains were recorded, with a median of 12 spike trains per participant (7.5, 22). The total number of motor units and test units per participant is described in Table 1.

**Table 1:** Total number and median (interquartile range) of motor units and test units for each group

Variable	AT	Control
Total number of motor units	128	111
Number of motor units per participant	11 (6.5, 16.5)	6 (3.7, 11.7)
Total number of test units	103	105
Number of test units per participant	5 (3.2, 7.0)	4 (1.7, 7.5)

Data represent median (25th, 75th percentile)

**Fig. 2:**
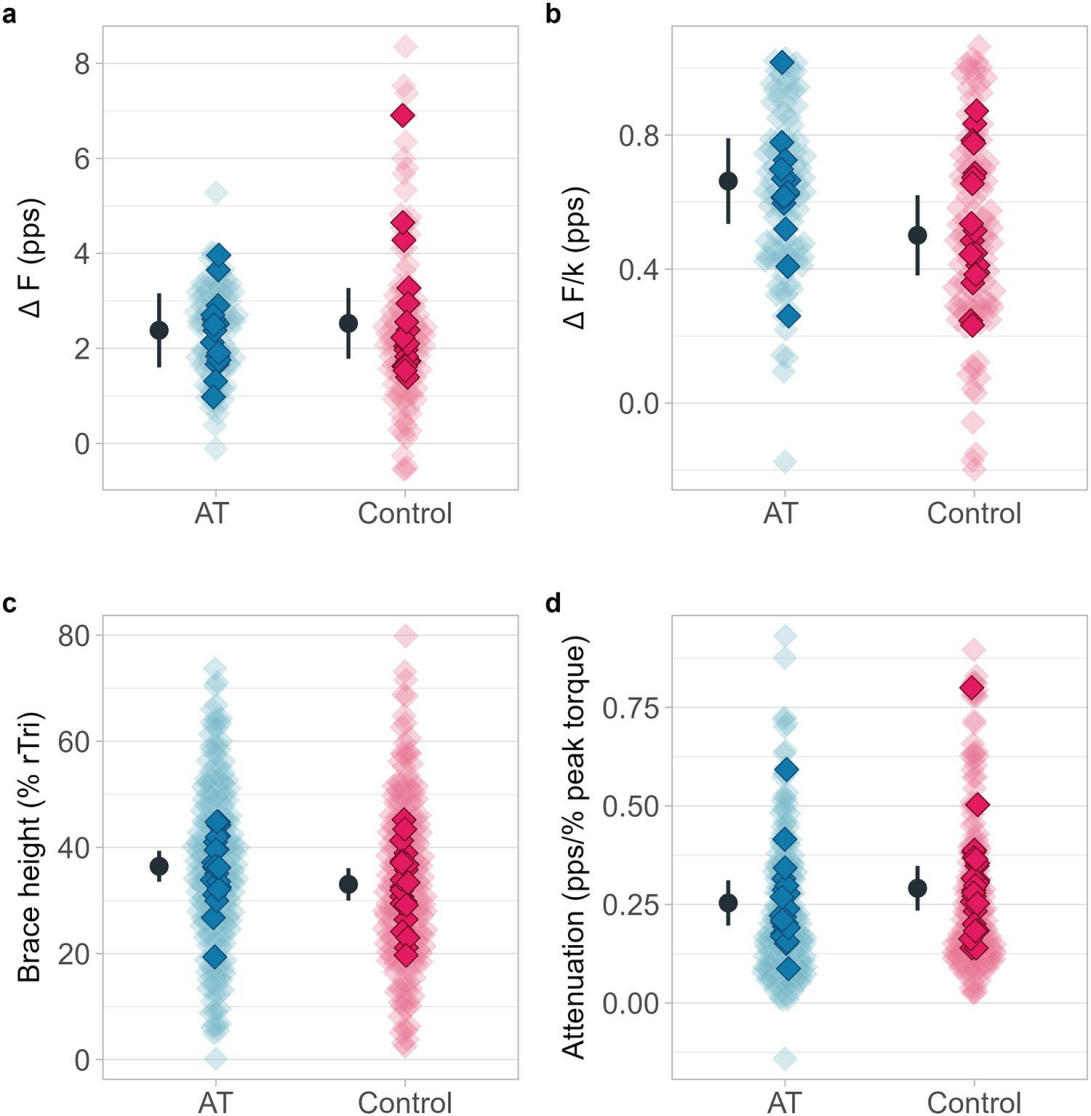
Δ*F* (**a**), Δ*F*/*k* (**b**), brace height (**c**) and Attenuation (**d**) for each group. Background diamonds represent all individual data points, whereas the darker diamonds represent each participants mean data point. Estimated marginal mean and 95% confidence intervals are offset to the left. *pps* pulse per second, *rTri* right triangle

**Fig. 3:**
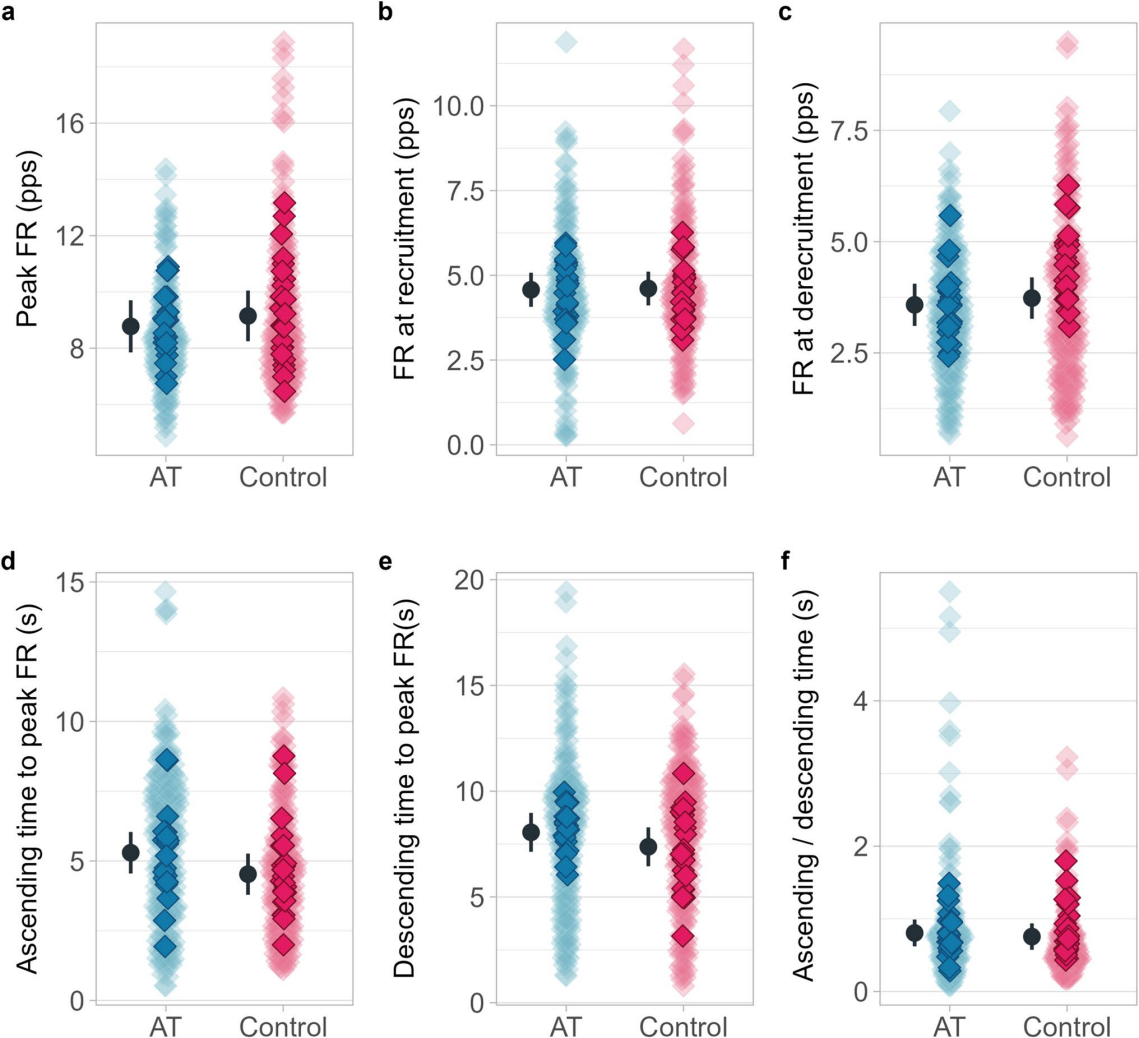
Peak FR (**a**), FR at recruitment (**b**), FR at de-recruitment (**c**), ascending time (**d**), ascending time (**e**) and ascending/descending time (**f**) for each group. Background lighter diamonds represent all individual data points, darker diamonds represent each participants mean data point. Estimated marginal mean and 95% confidence intervals are offset to the left. *pps* pulse per second, *s* seconds

There were no differences between groups for ΔF (*p* = 0.795, *F* = 0.070, Fig. 2A), and age did not influence the model (*p* = 0.928, *F* = 0.008). Similarly, no differences were found for Δ*F*/*k* (*p* = 0.09 *F* = 3.297, Fig. 2B), with no influence of age (*p* = 0.478, *F* = 0.533). Other measures showed no significant differences, including brace height (*p* = 0.07, *F* = 3.60, Fig. 2C) attenuation (*p* = 0.27, *F* = 1.32, Fig. 2D). Additionally, there were no significant differences for peak firing rate (*p* = 0.55, *F* = 0.359, Fig. 3A), firing rate at recruitment (*p* = 0.91, *F* = 0.011, Fig. 3B), firing rate at de-recruitment (*p* = 0.638, *F* = 0.226, Fig. 3C), ascending time from recruitment-to-peak firing rate (*p* = * p* = * p* = 0.14, *F* = 2.387, Fig. 3D), descending time from peak firing rate to de-recruitment (*p* = 0.28, *F* = 1.235, Fig. 3E), and the ascending/descending time ratio (*p* = 0.68, *F* = 0.165, Fig. 3F). Peak isometric torque was 115.1 Nm (95% CI 94–136.2) in the AT group and 110.9 Nm (88–133.0) in the Control group, with no significant difference between groups (*p* = 0.77). These results have been partially reported previously (Fernandes et al. 2021). In summary, our findings indicate that PICs and measures of neuromodulation of synaptic input, such as brace height, do not differ between runners with Achilles tendinopathy and healthy controls for low-threshold soleus motor neurons during submaximal contractions at 20% MVC. Estimated marginal means, mean differences and effect size are presented in Table 2.

**Table 2:** Δ*F*, Δ*F*/*k*, brace height, attenuation and motor unit firing rates characteristics, estimated marginal means and mean differences between AT and control groups

Variable	AT	Control	Mean difference (95% CI)	Hedges *g* (95% CI)
ΔF	2.24 (1.0–3.4)	2.47 (1.2–3.7)	− 0.23 (− 2.1 to 1.6)	0.07 (− 0.3 to 0.2)
Δ*F*/*k*	0.64 (0.4–0.8)	0.63 (0.4–0.8)	0.01 (− 0.3 to 0.3)	0.4 (0.1–0.7)
Brace height	36.8 (33.7–39.9)	32.8 (29.6–36.1)	3.9 (− 8.3 to 0.4)	0.2 (0.04–0.4)
Attenuation	0.41 (0.3–0.4)	0.44 (0.3–0.5)	− 0.04 (− 0.03 to 0.1)	− 0.2 (− 0.4 to − 0.07)
Peak firing rate	8.68 (7.6–9.7)	9.21 (8.2–10.2)	− 0.53 (− 2 to 0.9)	− 0.3 (− 0.5 to − 0.2)
Firing rate at recruitment	4.66 (4.1–5.2)	4.47 (3.9–5.1)	0.19 (− 0.5 to 0.9)	− 0.1 (− 0.3 to 0.06)
Firing rate at derecruitment	3.67 (3.1–4.2)	4.04 (3.5–4.6)	− 0.37 (− 1.1 to 0.4)	− 0.2 (− 0.3 to 0.01)
Ascending time	4.55 (3.7–5.3)	4.59 (3.8–5.3)	− 0.04 (− 1.1 to 1.0)	0.3 (0.2, 0.5)
Descending time	8.21 (7.1–9.3)	8.20 (7.1–9.3)	0.007 (− 1.5 to 1.5)	0.03 (− 0.2, 0.2)
Ascending/descending time	0.67 (0.5–0.8)	0.64 (0.5–0.8)	0.03 (− 0.2 to 0.2)	0.3 (0.08 0.4)

Data represent mean (95% confidence interval, lower and upper limits) for each variable and hedges' *g* effect size

## Discussion

This study used different metrics to estimate the contribution of PICs to motor neuron firing, as well as the neuromodulatory, and inhibitory effects of PICs (ΔF, brace height and attenuation slope) and different motor unit firing characteristics, from lower-threshold motor units of soleus, in runners with and without mid-portion AT. Contrary to our initial hypothesis, Δ*F*, Δ*F*/*k*, and peak firing rates of low-threshold soleus motor units were not altered in runners with AT during submaximal contractions at 20% MVC. However, our data do not provide insights into potential changes involving higher-threshold motor units, which might be recruited at higher contraction intensities. Similarly, the absence of differences between groups on brace height and attenuation slope indicates similar influence of neuro-modulation and localized inhibition on their soleus motor neuron firing patterns. Therefore, our data indicate that motor unit firing patterns were not different in lower-threshold soleus motor neurons of runners with AT compared to those without. A similar intrinsic motor neuron excitability between runners with or without AT possibly indicates that (i) other musculo-tendinous or neurophysiological mechanisms might be contributing to the reduced plantar flexor function (O’Neill et al. 2019; Fernandes et al. 2021) observed in AT, (ii) other muscles (e.g., gastrocnemii) might be affected, and (iii) the intrinsic excitability might only be affected at higher intensity contractions.

The suggestion that soleus is the most involved muscle in the deficits in plantar flexor torque in AT was made based on differences in torque measures between knee flexed and extended (O’Neill et al. 2019). However, this analysis is limited to torque output, and it neglects some neurophysiological and mechanical mechanisms that enable force production. Perhaps, the deficits in torque observed in this group are present in other muscles (i.e., gastrocnemius lateralis and/or medialis) rather than soleus. As individual muscles of the triceps surae receive independent neural drive (Hug et al. 2020), we can hypothesize that the impact from increased inhibitory output from increased intracortical inhibition (Fernandes et al. 2021) in runners with AT, may not be affecting all three muscles the same way. New evidence suggests lower contribution to plantar flexor force from the lateral gastrocnemius and increased from soleus (Crouzier et al. 2020). Additionally, study (Fernandes et al. November 2022) from our group also found a reduction in neural drive only to gastrocnemius in runners with AT compared to healthy runners during submaximal contractions.

Soleus motor unit peak firing rate and recruitment threshold also showed no differences between groups. A reduction in neural drive to the motor neuron would negatively impact soleus motor neuron (Trajano et al. 2020; Heckman et al. 2005). This would have been seen as reduced firing rate and force output; however, this was not observed in our study. Although recruitment thresholds were similar between groups, indicating a similar sample of low-threshold motor units, it remains possible that two distinct motor unit populations were analyzed rather than a single, homogeneous pool. It is possible that different results would have been observed during higher contraction intensities, which would require recruitment of higher-threshold motor units and higher firing rates. Future studies should try and use higher contraction intensities and try and replicate our studies in higher-threshold motor units. These findings support the hypothesis that the ability of the soleus to produce force might not be affected in AT as previously suggested in the literature (O’Neill et al. 2019). Future studies should investigate motor neuron excitability of each individual muscle of the triceps surae to understand if these muscle deficits (McAuliffe et al. 2019) widely reported in runners with AT are in fact muscle specific.

Chronic pain has also been suggested to affect muscle activation in other chronic tendinopathies. Examples include reduced corticospinal excitability and lower infraspinatus activation in individuals with chronic rotator cuff tendinopathy (Ngomo et al. 2015), increased corticospinal inhibition of the rectus femoris in volleyball athletes with patellar tendinopathy (Rio et al. 2015), and altered activation patterns in the hip abductors during walking (i.e., prolonged activation of gluteus minimus and gluteus medius), in individuals with gluteal tendinopathy. If soleus force production was affected in runners with AT, soleus Δ*F* would have been reduced. Our sample of participants demonstrated reduced plantarflexor endurance (Fernandes et al. 2021), but no differences in peak force levels. Perhaps the deficits in plantar flexor force observed (McAuliffe et al. 2019) in runners with AT might be a result of changes in motor neuron excitability not in soleus, but in gastrocnemius lateralis and/or medialis.

### Strengths and limitations

The HD-EMG technology used in this study provides reliable estimates of individual motor unit firing rates (Martinez-Valdes et al. 2016). However, it requires data to be collected during isometric contractions to allow accurate motor unit recording and decomposition. Therefore, we cannot extrapolate our results into dynamic tasks such as calf raises or running. For optimal motor unit identification and ∆*F* calculation, submaximal intensity is required due to technological limitations. A torque relative to 20% of each individual MVC, used in this study, is a commonly used target torque in PIC studies (Orssatto et al. 2021, 2022; Trajano et al. 2020; Gorassini et al. 2002; Hassan et al. 2020; Powers et al. 2008). This lower intensity contraction recruits lower-threshold motor units. Consequently, our findings cannot be generalized to higher-intensity contractions involving high-threshold motor units. However, submaximal Δ*F* measures correlate strongly with changes in maximal force, suggesting they remain a useful proxy for investigating the mechanisms underpinning maximal contraction performance (Orssatto et al. 2023; Martino et al. 2024; Mackay et al. 2023). Higher contraction intensities would require a recruitment of higher-threshold motor units and allow greater neuromodulation (Orssatto et al. 2021b), which could present different results. However, these measures are not possible. Perhaps the data obtained in low-level forces do not represent the motor neuron excitability when higher intensity contractions are required (e.g., higher intensity running). It is relevant to note that there were no differences in isometric peak torque between groups; this finding is important because reduction in motor neuron excitability (e.g., reduced PICs amplitudes) would typically result in reductions in force production (Orssatto et al. 2022, 2023, 2021b; Martino et al. 2024). However, the absence of differences in peak plantarflexor torque does not rule out the possibility of selective soleus weakness, as previously proposed in the literature (O’Neill et al. 2019), potentially compensated by other muscles within the triceps surae. Therefore, despite similar overall torque outputs, neural alterations specifically affecting the soleus muscle might still exist, underscoring the importance of directly investigating intrinsic neural mechanisms at the motor neuron level.

PICs are highly sensitive to inhibitory stimuli (Hyngstrom et al. 2008) and pain responses can stimulate descending inhibitory inputs and affect motor neuron excitability (Farina et al. 2004; Tucker et al. 2012). A recent study (Hug et al. 2024) demonstrated that experimental knee pain reduced motor unit firing rates in the vastus lateralis, as well as a decrease PICs amplitude, without changes in neuromodulatory drive (brace height and attenuation slope). Notably, these changes in firing rates and PICs were limited to the experimental pain condition, and did not persist afterward. In our study, participants report no pain during testing. However, it remains unclear how the pain associated with Achilles tendinopathy might influence motor neuron excitability during running and which muscles might be most affected.

## Conclusion

This study investigated soleus motor neuron intrinsic excitability by estimating PICs amplitude (i.e., ΔF), as well as neuromodulatory and inhibitory inputs as potential neurophysiological mechanism underpinning the motor function deficits reported in runners with chronic mid-portion AT. Our data suggest that runners with mid-portion AT do not present deficits in soleus motor neuron excitability or firing rates compared to healthy controls. Future studies should investigate other musculotendinous and neurophysiological mechanisms and potential alterations of intrinsic motor neuron excitability and its influence on motor unit firing rate of gastrocnemius lateralis and/or medialis to investigate the mechanisms underpinning muscle deficits reported in AT.

## Data Availability

The datasets generated during and/or analyzed during the current study are available in the GitHub repository, https://github.com/GabeFernandess/PICs_SOL_runners_AT.
